# Mutation in *utp15* Disrupts Vascular Patterning in a p53-Dependent Manner in Zebrafish Embryos

**DOI:** 10.1371/journal.pone.0025013

**Published:** 2011-09-20

**Authors:** Kevin Mouillesseaux, Jau-Nian Chen

**Affiliations:** 1 Department of Molecular, Cell, and Developmental Biology, University of California Los Angeles, Los Angeles, California, United States of America; 2 Molecular Biology Institute, University of California Los Angeles, Los Angeles, California, United States of America; 3 Jonsson Comprehensive Cancer Center, University of California Los Angeles, Los Angeles, California, United States of America; 4 Cardiovascular Research Laboratory, University of California Los Angeles, Los Angeles, California, United States of America; Hong Kong University of Science and Technology, China

## Abstract

**Background:**

Angiogenesis is the process by which the highly branched and functional vasculature arises from the major vessels, providing developing tissues with nutrients, oxygen, and removing metabolic waste. During embryogenesis, vascular patterning is dependent on a tightly regulated balance between pro- and anti-angiogenic signals, and failure of angiogenesis leads to embryonic lethality. Using the zebrafish as a model organism, we sought to identify genes that influence normal vascular patterning.

**Methodology and Principal Findings:**

In a forward genetic screen, we identified mutant *LA1908*, which manifests massive apoptosis during early embryogenesis, abnormal expression of several markers of arterial-venous specification, delayed angiogenic sprouting of the intersegmental vessels (ISV), and malformation of the caudal vein plexus (CVP), indicating a critical role for *LA1908* in cell survival and angiogenesis. Genetic mapping and sequencing identified a G to A transition in the splice site preceding exon 11 of *utp15* in *LA1908* mutant embryos. Overexpression of wild type *utp15* mRNA suppresses all observed mutant phenotypes, demonstrating a causative relationship between *utp15* and *LA1908*. Furthermore, we found that injecting morpholino oligonucleotides inhibiting *p53* translation prevents cell death and rescues the vascular abnormalities, indicating that p53 is downstream of Utp15 deficiency in mediating the *LA1908* phenotypes.

**Conclusions and Significance:**

Taken together, our data demonstrate an early embryonic effect of Utp15 deficiency on cell survival and the normal patterning of the vasculature and highlight an anti-angiogenic role of p53 in developing embryos.

## Introduction

The correct patterning and function of the vascular system is essential to provide necessary nutrients, oxygen, and growth cues to perfused tissues, and remove metabolic waste in vertebrate organisms. During development, failure to form a properly functioning vasculature is embryonic lethal, complicating attempts to study this process *in vivo*. The zebrafish is an exceptional model organism for the study of early vascular development because it is amenable to genetic and embryological manipulations. In addition, zebrafish embryos continue to grow and develop for several days in the absence of the vasculature or blood circulation, providing a valuable tool to investigate the progression of vascular defects [1], [2].

In zebrafish, angioblasts, precursors of the endothelial cells (ECs) that make up the inner lining of the vasculature, arise in the lateral plate mesoderm during early somitogenesis [3]. During the process of vasculogenesis, the primitive ECs migrate to the midline, coalesce into a vascular cord by the 16 somite-stage (ss) [3], and eventually segregate into the dorsal aorta (DA) and posterior cardinal vein (PCV) through a cell-sorting process regulated by EphB4a-ephrinB2a, PI3K, and Notch signaling [4]. Subsequent expansion of the vascular network from the axial vessels is governed by angiogenesis. Angiogenesis in the zebrafish has been studied almost exclusively in the context of ISV formation [5]. Primary ISV sprouting initiates bilaterally from the DA beginning at approximately 20 hours post fertilization (hpf). The sprout then migrates dorsally to the dorsolateral roof of the neural tube, where the leading cell branches anteriorly and posteriorly to form the dorsal longitudinal anastomotic vessel (DLAV). ISV angiogenesis is primarily regulated by VEGF, as knockdown of VEGF by antisense morpholinos or mutation in the VEGF receptor 2 functional ortholog, *kdrl*, disrupt normal ISV formation [6]–[7], [8]. Additional signaling events are required to properly pattern the embryonic vasculature. For example, induction of Dll4 by VEGF in the leading tip cell of ISVs activates Notch signaling and inhibits sprouting in neighboring cells, allowing for proper migration of the nascent vessel [9], [10]. ISV migration is further restricted to somite boundaries by repulsive interactions between plexinD1 within the ECs and type 3 semaphorins expressed in the developing somites [11].

Complementary to studies of ISV formation, development of the caudal vein plexus (CVP) provides a venous-specific angiogenesis model in zebrafish [12]. The CVP forms via a very active period of angiogenic sprouting, beginning at 25 hpf when venous ECs of the PCV sprout and migrate ventrally, then fuse with neighboring tip cells. This process reiterates during a five-hour window, forming a primordial plexus by 30 hpf. By two days of development, the primitive CVP has matured into a complex, well-perfused, venous vascular network. Although the pro-angiogenic signals governing sprouting and patterning of the plexus are still being investigated, roles for Bmp signaling, protein geranylgeranylation, and Rho signaling in promoting the migration of venous ECs have been established in this system [12], [13].

The tumor suppressor gene, p53, plays a central role in controlling cell cycle and genome stability [14]. Activation of p53 often results in programmed cell death in response to acute cell stress [14], [15]. Genetic studies in the zebrafish and mouse models provide strong correlation between inactivation of essential genes, p53 activity, and lethal phenotype [16]–[17], [18], [19], [20], [21]. In addition to its role in apoptosis, p53 has been directly linked to regulation of angiogenesis by inducing thrombospondin-1 (Thbs1) [22], [23]. Thbs1 exhibits anti-angiogenic functions through various mechanisms [24], [25]. It has been demonstrated that Thbs1 inhibits EC migration by activating cell-surface proteins such as CD36 and β1 integrins through direct binding [25]–[26], [27]. In addition, Thbs1 could prevent the matrix remodeling necessary for EC migration by inhibiting the matrix metalloprotease (MMP) 3-mediated cleavage of pro-MMP9 to its active form [24]. Furthermore, Thbs1 competes with angiogenic growth factors by binding to extracellular proteoglycans, thereby reducing their bio-availability [26]. While a strong induction of Thbs1 expression by p53 has been noted *in vitro* [22], [23], the possible impact of p53 upregulation on developmental angiogenesis remains to be investigated.

In this report, we describe the isolation of zebrafish mutant *LA1908* from an ENU mutagenesis screen based on the presence of a severely deformed CVP, delayed ISV formation, and significant cell death in the central nervous system. We also noted that several markers of arterial or venous endothelium are mis-expressed and the expression of *thbs1* was significantly upregulated in *LA1908* mutants. Positional cloning identified a point mutation in the *utp15* locus leading to the production of three splice variants in *LA1908*. Utp15 is a U3 snoRNA-associated protein of the Small Subunit Processome (SSU) [28], essential for 18S rRNA biogenesis. In yeast, loss of any one of the U3 proteins resulted in 18S rRNA biogenesis defects and disrupts formation of the 40S small ribosomal subunit [28], but their roles in vertebrate morphogenesis have not been fully investigated. Injection of wild type *utp15* mRNA was sufficient to rescue all observed defects in *LA1908* mutants, demonstrating a causative relationship between *utp15* deficiency and the vascular and cell survival defects observed in *LA1908*. More interestingly, blocking p53 activity in *LA1908* completely suppressed apoptosis and rescued the ISV and CVP defects, indicating that p53 functions downstream of the loss of Utp15 in these embryos. Finally, the abnormally enhanced *thbs1* expression was suppressed by the knockdown of p53 in a dose-dependent manner. Taken together, our data provide the first genetic evidence for a p53-mediated anti-angiogenic effect of *utp15* deficiency during development.

## Results

### *LA1908* exhibits defects in developmental angiogenesis

In an effort to identify genes important for cardiovascular development, we performed an ENU mutagenesis screen in Tg(*kdrl*:GFP) zebrafish. *LA1908* was identified from this screen on the basis of severe vascular abnormalities at 48 hours post fertilization (hpf). We utilized confocal microscopy to analyze vascular development and found that angiogenesis in both the trunk and the tail regions is abnormal.

**Figure 1:**
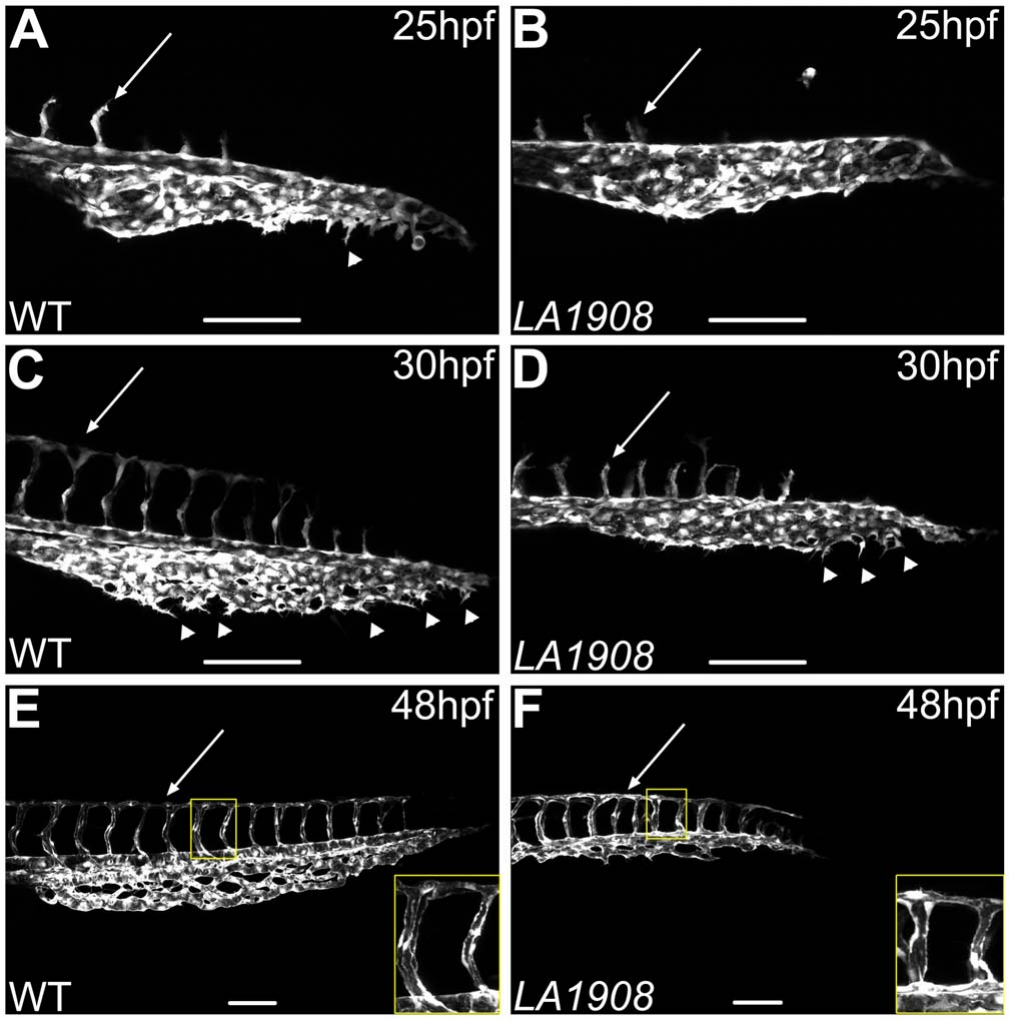
Confocal microscopic analysis of angiogenesis in *LA1908* embryos. A–F, Confocal z-stack projection images were captured of the trunk and tail vasculature of wild type (A, C, E) and *LA1908* mutant (B, D, F) embryos at 25 (A, B), 30 (C, D), or 48 hpf (E, F). CVP endothelial tip cells are indicated by arrowheads; extent of ISV maturation is indicated by arrows in A–D. Delay of primary ISV sprouting is most prominent at 30 hpf (C and D, arrows), when WT ISVs have reached their dorsal terminus and branched to form the DLAV (C), while mutant ISVs remain stunted (D). By 48 hpf, ISV number is equivalent between WT and mutant embryos and DLAV has formed in both groups (E, F, arrows). Defects in CVP angiogenesis result in a thinner and shorter plexus that lacks the complexity of WT embryos (F versus E). Insets in E and F, enlarged regions indicated by yellow rectangles, showing ISV lumens. Scale bars are 100 µm.

In wildtype (WT) embryos, the primary ISV sprouts emerge from the DA during the segmentation stage and migrate to approximately the level of the dorsolateral roof of the neural tube and branch to form the DLAV by 30 hpf (Figure 1C) [5]. We noted a reduced number of primary ISVs in *LA1908* at 25 hpf (Figure 1A, B and S1F). Mutant ISVs are significantly shorter and fail to reach the dorsal branching point, preventing formation of the DLAV in *LA1908* at 30 hpf (Figure 1D). Interestingly, the ISV defects recover by 48 hpf. At this time, *LA1908* embryos have a similar number of ISVs as their WT siblings (data not shown). These ISVs are patent, regularly spaced, have branched to form the DLAV (Figure 1E, F), and emerge from both the DA and PCV (Figure S2), suggesting a delay in ISV angiogenesis. To investigate if the delay in ISV angiogenesis was a consequence of general developmental delay in *LA1908*, we examined somite number and other morphological parameters commonly used to stage zebrafish embryos [29] and found no differences between *LA1908* and their WT siblings (Figure S1E). These findings indicate that the vascular defects observed in *LA1908* are likely the result of a specific delay in vessel formation and demonstrate an effect of *LA1908* on the proper timing of angiogenesis in zebrafish.

In addition, formation of the CVP is markedly defective in *LA1908* embryos. We have previously shown that venous ECs of the PCV undergo an active angiogenic phase beginning at approximately 25 hpf (Figure 1A and Movie S1) and develop a primitive plexus by 30 hpf (Figure 1C) [12]. Time-lapse confocal images indicate that, while tip cells are able to emerge from the venous endothelium in *LA1908* (Figure 1D), these cells quickly retract and never develop into mature, migrating ECs (Movie S2). In contrast to the ISVs, the CVP shows no capacity to recover in *LA1908* at later developmental stages, resulting in failure to form a complex plexus in the ventral tail region (Figure 1F).

### Abnormal vascular gene expression in *LA1908*

**Figure 2:**
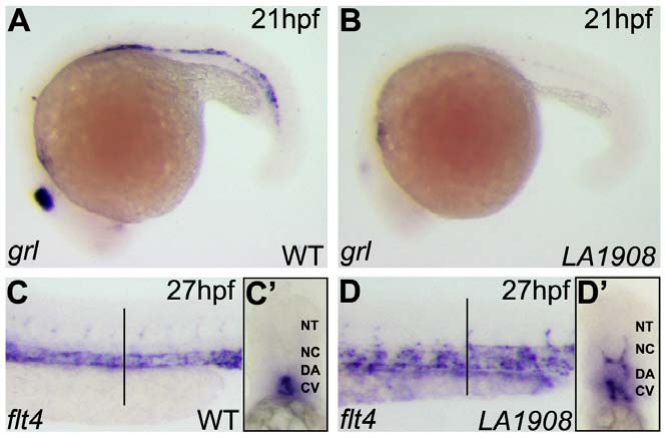
Aberrant expression of arterial and venous endothelial cell markers in *LA1908* mutant embryos. A–B, Expression of *grl*, an arterial EC marker, was dramatically reduced in *LA1908* mutant embryos (B versus A) at 21 hpf. C–D, Expression of the venous marker, *flt4,* was increased and localized ectopically in *LA1908* mutant embryos at 27 hpf (D versus C). Vertical black bars, approximate location of vibratome cross-sections shown in C’–D’. C’–D’, Vibratome cross-section of embryos shown in C, D, revealing *flt4* expression was appropriately restricted to the CV in 27 hpf wildtype embryos (C’), but is ectopically expressed in the DA and ISVs in *LA1908* mutant embryos (D’). Embryos shown are representative from triplicate experiments performed on a minimum of 5 embryos per condition.

We further characterized the nature of the vascular abnormalities by examining the expression of markers of arterial and venous identity. The expression of the arterial marker, *grl,* was significantly reduced or absent in mutant embryos (Figure 2A, B). Conversely, the expression of *flt4*, a marker of venous differentiation, persists in the DA and the ISVs (Figure 2C–D’). These data suggest that *LA1908* perturbs the normal pattern of arterial-venous gene expression and may disrupt the expression of angiogenic regulatory genes. However, pro-angiogenic factors such as VEGF and BMPs are expressed normally in mutant embryos (Figure S3), and the expression of neural (*krox20*) and hematopoetic (*cmyb*) differentiation markers is indistinguishable between WT and mutant embryos (Figure S1 A–D), suggesting that *LA1908* does not disrupt global gene expression, but rather regulates specific subsets of vascular genes.

### *LA1908* manifests extensive apoptosis during somitogenesis

**Figure 3:**
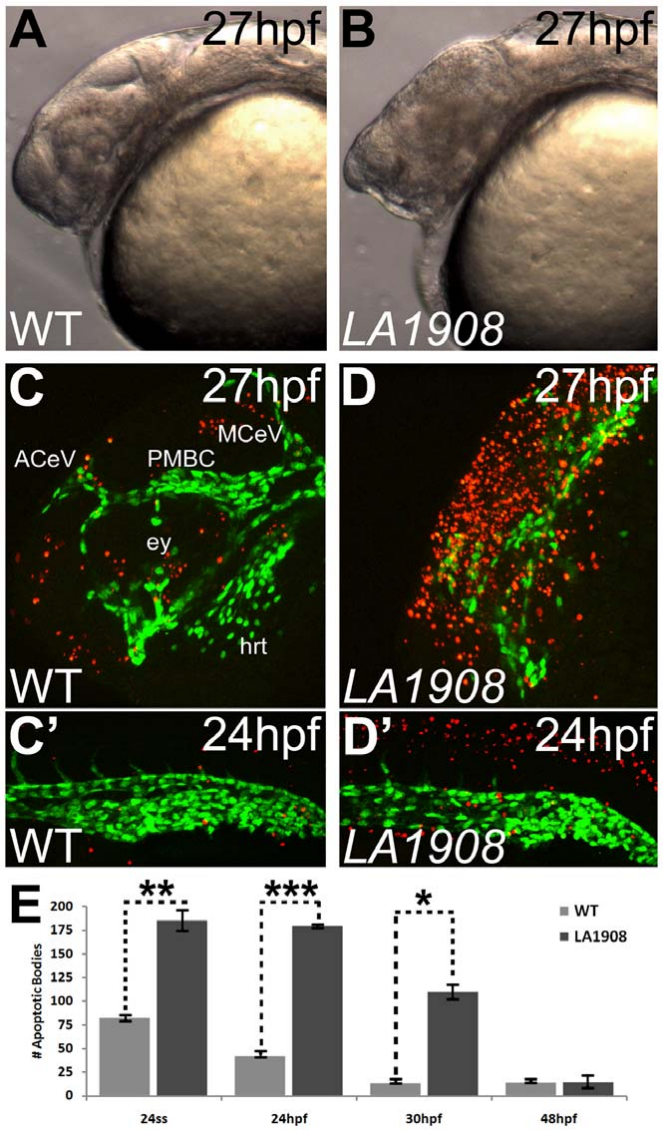
Apoptosis is pervasive in neural tissues of *LA1908* mutant embryos. A–B, Brightfield phase-contrast imaging of mutant embryos revealed craniofacial disorganization and extensive cell death (hazy dark brown appearance in midbrain in B versus A). C–D’, Apoptosis is significantly induced in *LA1908* mutant embryos (D, D’) relative to stage-matched WT siblings (C, C’). Green fluorescence marks the endothelial cells. Red fluorescence indicates apoptotic cells detected by TUNEL assay. ACeV, Anterior cerebral vein. ey, eye primordium. hrt, embryonic heart. MCeV, Middle cerebral vein. PMBC, Primordial midbrain channel. E, Quantitative and statistical analyses of apoptotic bodies at several stages of development in WT versus mutant embryos. * = p-value<0.05, ** = p-value<0.005, *** = p-value<0.00005 by two-tailed t-test, n = 3 embryos for each condition, data are average±SEM.

In addition to the vascular defects described above, we observed a significant level of cell death in the CNS during segmentation stages in *LA1908* mutant embryos, leading to the formation of smaller heads and eyes (Figure 3A, B). To determine if the cell death was apoptotic in nature, we performed a whole-mount, TUNEL-based, fluorescent apoptosis assay and quantified the results via z-stack confocal microscopy. The number of apoptotic cells was significantly upregulated in *LA1908* embryos compared to WT siblings, and apoptotic bodies were largely concentrated in neural tissues of the head and neural tube (Figure 3C–E and data not shown). Although some apoptotic ECs were observed in the trunk and ventral tail region of *LA1908* mutant embryos, the number of apoptotic ECs was not significantly different than that observed in WT siblings. The apoptotic phenotype dissipated by 48 hpf and no significant differences in numbers of apoptotic cells were observed in 2-day-old WT and mutant embryos (Figure 3E). This suggests *LA1908* plays an important role in promoting cell survival during early embryogenesis.

### *LA1908* encodes a G to A transition in the splice acceptor site preceding exon11 of *utp15*

**Figure 4:**
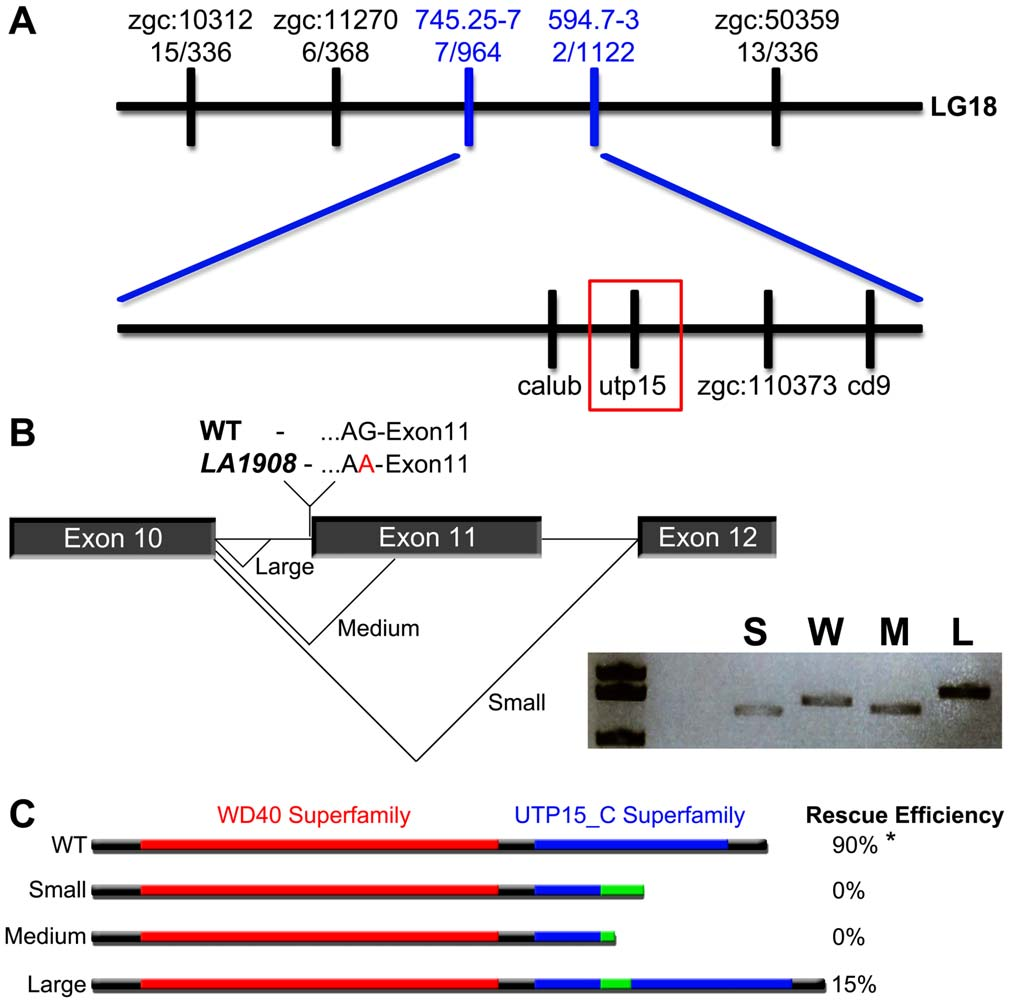
The *LA1908* locus encodes zebrafish *utp15*. A, Positional cloning showed tight linkage between *LA1908* and *utp15*. B, Sequence analysis of the *utp15* gene revealed an intronic G to A point mutation, abolishing the splice acceptor site preceding exon11 and resulting in three *utp15* cryptic splicing variants; a 134 bp deletion (*utp15 small*), a 26 bp deletion (*utp15 medium*), and a 36 bp insertion (*utp15 large*). Inset in (B) shows the relative cDNA sizes of S (*utp15 small*), W (wild type *utp15*), M (*utp15 medium*), and L (*utp15 large*) variants. C, The protein structure and translational consequences of *utp15* splice variants. Utp15 small and medium proteins contain premature stop codons resulting in truncated proteins. Injecting *in vitro* synthesized mRNA generated from these constructs was unable to rescue *LA1908* mutant phenotype. Utp15 large contains a 12 amino acid insertion and displays some ability to rescue phenotype. Red bar, WD40 protein-protein interaction domain. Blue bar, utp15 C-terminal superfamily domain of unknown function. Green bar, altered peptide sequence resulting from cryptic splicing. * = p-value <0.001 by Chi-Squared analysis of Mendelian ratios of a minimum of 100 embryos per condition. Rescue efficiency is calculated as # embryos exhibiting *LA1908* mutant phenotype/# total embryos with *LA1908* mutant genotype, n = 10 (WT), 28 (small), 11 (medium), and 13 (large) mRNA injected mutant embryos.

We performed positional cloning to identify the molecular lesion of *LA1908*. Whole-genome scanning identified genetic linkage between *LA1908* and Z9941 placing the locus on chromosome 18. Subsequent genotyping of individual mutant embryos revealed Z9941 was 29.4 cM from the locus. Fine mapping using additional microsatellite markers further narrowed the interval to 0.7 cM upstream (7 recombinant chromosomes in 964 meioses) and 0.1 cM downstream (2 recombinant chromosomes in 1122 meioses) (Figure 4A) which encompasses four genes (*calub, utp15, zgc:110373, cd9*). Sequencing of the coding regions of these genes did not identify non-synonymous point mutations. However, sequence analysis of introns revealed a G to A transition in the splice acceptor site of intron 10 preceding exon 11 of *utp15* (Figure 4B), resulting in the use of alternative AG sites preceding exon 12, within exon 11, and in intron 10, generating three cryptic splice variants named *utp15 small, utp15 medium,* and *utp15 large* (Figure 4B). Both *utp15 small* and *utp15 medium* cause frame-shifts and produce truncated Utp15 proteins, while *utp15 large* causes a 12 amino acid in-frame insertion in the UTP15 C-terminal superfamily domain (Figure 4C).

We injected wild type *utp15* mRNA (WT Utp15) into *LA1908* mutant embryos at the 1-cell stage and found that 90% of WT Utp15-injected mutant embryos developed normally and were indistinguishable from their WT siblings (Figure 4C, n = 221), demonstrating a causative relationship between the *utp15* mutation and the *LA1908* phenotype. Neither Utp15 small nor Utp15 medium were able to rescue the *LA1908* phenotype (Figure 4C, n = 102 and 136, respectively), indicating that the truncated proteins encoded by these splice variants are non-functional. Utp15 large, on the other hand, displayed a slight ability to rescue the mutant phenotype (15%, n = 109) (Figure 4C), suggesting that the 12aa insertion does not completely abolish the function of Utp15 and that *LA1908* might be a hypomorphic allele.

### *utp15* is expressed ubiquitously in early embryos, with later expression enriched in neural tissues and the vasculature

**Figure 5:**
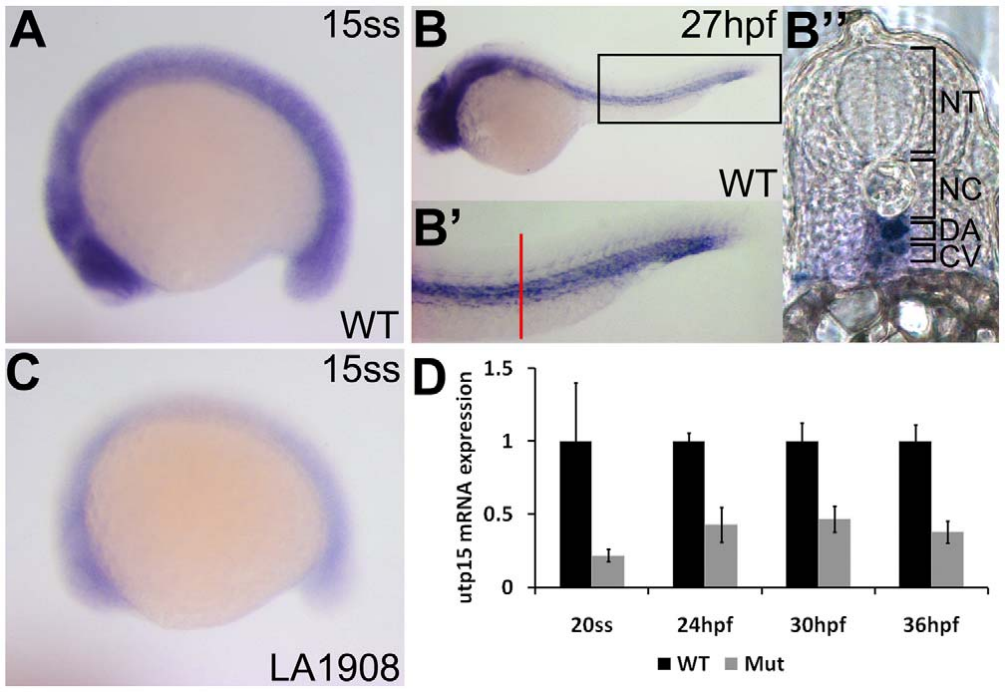
Analysis of endogenous *utp15* expression by whole-mount in situ hybridization. A, *utp15* is expressed ubiquitously in developing embryos at early stages. B, Expression was restricted to neural and vascular tissues after one day of development. B’, 2x magnification of the rectangular region indicated in (B), showing expression in the dorsal aorta and cardinal vein. Red bar, approximate location of the vibratome cross-section shown in (B’’). C–D, *utp15* was dramatically reduced in mutant embryos, as shown by in situ hybridization (C) and qRT-PCR (D) analyses. A–C, Embryos shown are representative of triplicate experiments performed on a minimum of 5 embryos per condition. D, Data shown are the average fold changes in expression±SEM from two replicate qRT-PCR assays using pooled RNA from 20 embryos per condition.

We next utilized whole mount in situ hybridization to examine the expression of *utp15* mRNA in WT and mutant embryos. We discovered that *utp15* is ubiquitously expressed in WT embryos up to the segmentation stage (Figure 5A) and becomes restricted to the axial vasculature of the trunk and tail as well as to neural tissues of the CNS in 1-day-old embryos (Figure 5B-B’’). The enrichment of *utp15* expression in the vasculature after 1 day of development correlates with the vascular patterning and endothelial cell gene expression defects observed in *utp15^LA1908^*. Furthermore, whole mount in situ hybridization and quantitative RT-PCR analyses in stage-matched mutant siblings showed that total *utp15* mRNA levels are drastically reduced in mutant embryos, indicating that the mutant splice variants are unstable (Figure 5C, D).

### Phenotype in *LA1908* is dependent on activation of p53

**Figure 6:**
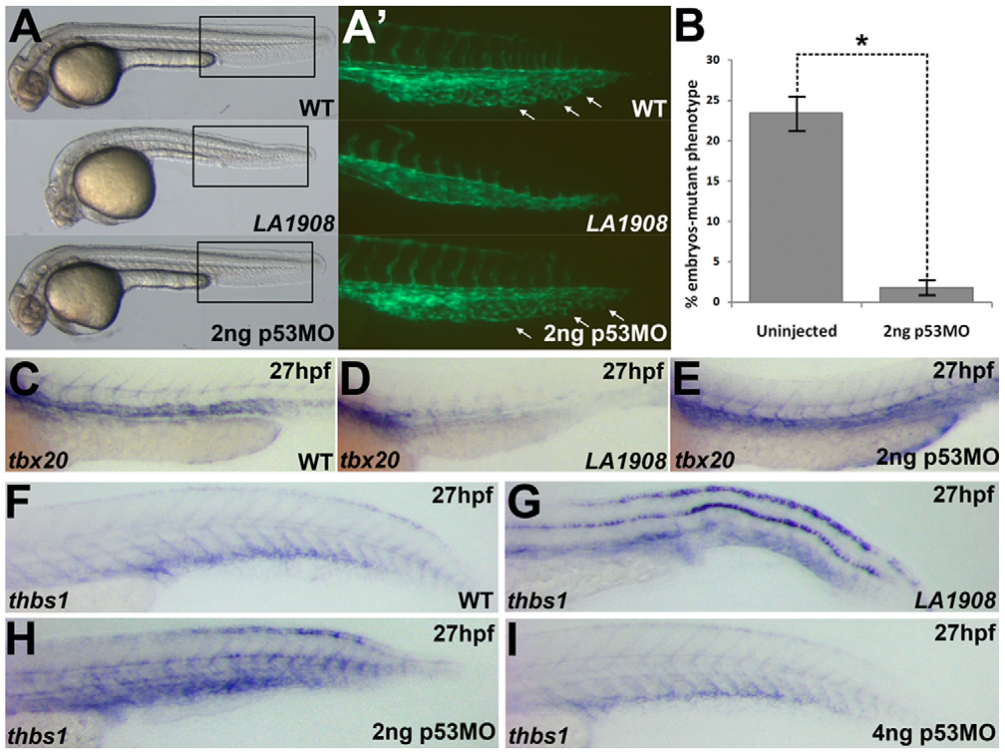
Loss of function of *utp15* induces p53 activity. A, Brightfield Phase contrast images of 30 hpf embryos. *LA1908* mutant embryos manifest defects in the brain and the vasculature as well as a significantly shorter body when compared to their wild type siblings. Injection of 2 ng p53MO is sufficient to prevent CNS necrosis (A), restore normal CVP morphology (A’), and normalize the gross morphology of the mutant embryos. A’, Magnified image of boxed regions of Tg[*kdrl*:GFP] embryos in (A). Note normal ISV patterning and presence of EC sprouting within the CVP (white arrows), resulting in a prototypical plexus. B, Chi-squared analysis of WT:Mutant ratios from 3 independently replicated experiments. n = 220 uninjected control embryos, 298 2 ng p53MO-injected embryos, * = p-value<0.001. Data are average +/- SEM. C–E, Analysis of *tbx20* expression, a marker of arterial endothelial cells, demonstrated p53MO also restores normal expression levels and patterning (E) which were lost in mutants (D) relative to WT (C) embryos. Embryos are representative of experiments performed in triplicate on a minimum of 5 embryos per condition. F–H, *thbs1* is expressed in a p53-dependent manner. Under normal conditions (F), expression is observed in the floor plate, dorsal neural tube, and in the ventral somites/axial vasculature. In mutant embryos, *thbs1* expression is substantially upregulated (G). Knockdown of p53 reduced *thbs1* expression in a dose-dependent manner. Roughly half (18/41) of the embryos injected with 2 ng p53MO have *thbs1* levels as shown in (H), with the remainder (23/41) displaying WT (F) levels. When the dose is increased to 4 ng (I), 82% of embryos (31/38) had WT (F) levels of *thbs1* mRNA, while only 18% (7/38) appeared as in (H).

The activation of p53 plays a key role in cell survival, and *utp15^LA1908^* mutant embryos manifest massive programmed cell death, raising a possibility that p53 may mediate the effect of loss of Utp15 during early embryogenesis. Consistent with this hypothesis, expression of p53 was strongly induced in all tissues of mutant embryos (Figure S4). We further tested whether the phenotypes of *utp15^LA1908^* mutant embryos is indeed dependent on p53 and found that blocking p53 activity using antisense morpholino oligonucleotides restored gross wild type appearance in *utp15^LA1908^* embryos (Figure 6A, B, n = 298). Not only is apoptosis suppressed in early embryogenesis, but the formation of the ISVs and the CVP (Figure 6A’, B) are normalized in embryos deficient in both Utp15 and p53. Similarly, the expression patterns of arterial and venous markers, such as *tbx20* (Figure 6C–E), *grl*, *flt4*, *ephB4a*, *aplnra*, and *dab2* (data not shown) in p53 morpholino-injected *utp15^LA1908^* mutant embryos resemble those in WT embryos. Furthermore, we noted a significant upregulation of *thbs1*, a potent matricellular angiogenesis inhibitor, in *LA1908* (Figure 6F, G). Blocking p53 activity down-regulates *thbs1* expression in a dosage-dependent manner (Figure 6G–I). Taken together, these findings indicate a p53-dependent effect of Utp15 deficiency during zebrafish development.

## Discussion

Utp15 is a U3 snoRNA-associated protein involved in the cleavage of pre-ribosomal RNA. Depletion of Utp proteins affects the production of 18S rRNA in yeast [28]. The requirements for Utp proteins during development is elusive, but the findings that zebrafish insertional mutations perturbing *utp15* and *utp11-like* genes lead to embryonic lethality [17] indicate an essential role for Utp proteins in vertebrate embryogenesis. Here we report the isolation of *utp15^LA1908^*, a zebrafish mutant carrying a point mutation affecting the splicing of *utp15*. Similar to the insertional mutant, *utp15^hi1370Tg^*, *utp15^LA1908^* is embryonic lethal. In addition, we find that embryos deficient in *utp15* manifest massive apoptosis during the first day of development. Starting from the second day of development, the number of apoptotic cells decreases in *utp15^LA1908^*, and no significant differences in the number of apoptotic bodies are observed between mutant embryos and their WT siblings by 48 hpf, indicating a requirement for Utp15 in cell survival during early embryogenesis. We further found that blocking p53 activity is sufficient to suppress apoptosis in *utp15^LA1908^*. This is very similar to results obtained in the zebrafish *bap28* mutant. BAP28 shares low similarity to yeast *utp10*, and its activity is required for the biogenesis of rRNA in zebrafish [30]. Loss-of-function of *bap28* in zebrafish induces p53-dependent apoptosis in the CNS [30]. Phenotypic similarities between *utp15* and *bap28* suggest a p53-dependent cell survival mechanism in response to ribosomal biogenesis stress. This idea is strongly supported by previous studies in cultured mammalian cells, which demonstrated that p53 activity is sensitive to errors in rRNA biogenesis [19].

The roles for Utp proteins in morphogenesis have not been investigated. The defects we observe in *utp15^LA1908^*, including the altered timing of ISV formation and perturbed morphogenesis of the CVP as well as the mis-expression of arterial and venous markers, demonstrate a previously unappreciated role for Utp proteins in angiogenesis. Mechanisms by which Utp15 deficiency perturbs angiogenesis are unclear. It is possible that massive apoptosis during segmentation stages induces systemic inflammatory responses in the developing zebrafish and thereby affects normal angiogenesis in a non-cell autonomous manner. Alternatively, loss of function of Utp15 may directly influence the formation of embryonic vasculature. The expression of *utp15* becomes enriched in the vasculature after one day of development, which coincides with the appearance of vascular defects in *utp15^LA1908^* embryos and suggests that *utp15* may also have cell autonomous roles in later vascular developmental processes. Regardless of whether Utp15 influences embryonic anigogenesis through a cell-autonomous or a non-cell autonomous mechanism, the finding that blocking p53 activity in *utp15^LA1908^* mutant embryos could suppress the vascular phenotypes suggests that the effect of Utp15 deficiency on angiogenesis is mediated by p53. Inhibitory effects of p53 on angiogenesis via repressing the production of VEGF or promoting the production of anti-angiogenic factors such as Thbs-1 have previously been noted [22], [23], [31], [32]. Interestingly, the expression of *thbs1* is upregulated in tissues adjacent to the ISVs and CVP in *utp15^LA1908^* embryos. Knocking down p53 activity eliminated the upregulation of *thbs1* and rescued the angiogenesis defects in *utp15^LA1908^*, suggesting that disruption of ribosomal biogenesis induces a p53-Thbs1-mediated anti-angiogenic pathway.

In this study, we noted that the delay of ISV formation recovered by two days of development, but the defect in CVP formation was permanent in *utp15^LA1908^* embryos. Mechanisms underlying the disparate impact of Utp15 deficiency on ISV and CVP formation require further investigation. One attractive possibility is the potential of distinct pro-angiogenic signals mediating ISV versus CVP formation and that signals governing ISV sprouting are stable during early development whereas the growth cues for the CVP are transient. The significance of signaling from the somite-produced VEGF ligand to the VEGFR2/*kdrl* receptors on the endothelial cells in ISV formation has been firmly established [6], [8], [9], [33], whereas emerging evidence suggests that BMPs in the ventral tail mesenchyme promote caudal vein plexus formation [13]. Interestingly, we found that the production of VEGF in the ventral somites persists through embryonic day 2 (Figure S2), while expression of the BMPs 2b and 4 in the ventral tail is high at the onset of CVP angiogenesis (Figure S2) but fades away soon afterwards (Figure S2). The dynamic expression patterns of VEGF and BMPs are consistent with our postulation that a stable pro-angiogenic signal such as VEGF allows the recovery of ISV formation whereas transient signals such as BMPs prevent the CVP formation at later developmental stages in *utp15^LA1908^* embryos. Further analysis of the relationship between VEGF and BMP signaling and ISV and CVP angiogenesis will help clarify the dynamic regulatory mechanisms of angiogenesis in different vascular beds in the zebrafish embryo.

## Materials and Methods

### Zebrafish Husbandry

*LA1908* was isolated from F2 intercrosses of Tg(*kdrl*:GFP)*^LA116^* zebrafish mutagenized by treatment with ENU according to standard protocols [34], [35]. Fish and embryos were maintained as described previously [29].

### Confocal Microscopy

Confocal Z-stack images of *utp15^LA1908^* embryos and their wild type siblings were captured using a Zeiss LSM510 epifluorescent laser scanning microscope equipped with an Achroplan 20x/0.5W water immersion objective. Z-stack projections were created using Zeiss LSM Image Browser Software (ver. 4.2.0.121, Carl Zeiss, Inc.). Live embryos were anesthetized in 0.01% tricaine and both live and fixed embryos were embedded laterally in 1% agarose.

### Whole-Mount *In Situ* Hybridization

Embryos for in situ hybridization were raised in embryo medium supplemented with 0.2 mM 1-phenyl-2-thiourea to maintain optical transparency [29]. Whole-mount in situ hybridization was performed as described previously [36]. Briefly, embryos were fixed in 4% paraformaldehyde in phosphate-buffered saline (PBS), followed by dehydration in methanol. Embryos were digested with proteinase K and then pre-incubated in hybridization buffer (65% formamide, 5X SSC, 5mg/ml torula yeast RNA, 50 µg/ml heparin, 0.1% Tween-20), then hybridized with digoxigenin-labeled antisense probes overnight at 67°C. The antisense RNA probes used in this study include *grl, flt4, ephB4a, ephrinB2a, kdrl, krox20, cmyb, utp15, p53, tbx20, dab2,* and *aplnra*. Embryos were washed in solutions of decreasing osmolarity and then blocked in 5% goat serum, 2mg/ml BSA in PBT (PBS+0.1% Tween-20). Labeled probes were detected using an alkaline phosphatase-conjugated anti-digoxigenin antibody followed by reaction in the presence of nitro-blue tetrazolium and 5-Bromo-4-chloro-3-indolyl phosphate. Stained embryos were mounted in 3.25% methylcellulose and imaged on a Zeiss Stemi SV11 Apo stereomicroscope using AxioVision software (ver. 4.8.1.0, Carl Zeiss, Inc.) under direct lighting.

### Vibrating microtome sectioning of embryos stained by whole-mount in situ hybridization

Embryos stained by whole mount in situ hybridization were mounted tail-down in 4% (W/V) low-melt agarose (Fisher) dissolved in E3. An agar plug containing the embedded embryos was cut and affixed to the tray of a 1000 Plus Sectioning System (The Vibratome Company). 100 µm sections were cut into ice-cold PBS at speed = 5 and amplitude = 4. Sections were mounted onto standard laboratory slides and imaged on a Zeiss Axioplan2 compound microscope using automatic ExtendedFocus ™ and a 20x/0.50 Ph.2 objective.

### Positional Cloning

Whole-genome scanning for genetic linkage was performed by PCR amplification of microsatellite repeats from pooled WT versus mutant genomic DNA (gDNA) samples using published markers [37]. gDNA was extracted from individual methanol-fixed embryos at 55° overnight in 50 µL lysis buffer composed of proteinaseK at 100 µg/mL, 10 mM Tris pH 8.0, 2 mM EDTA, and 0.2% (V/V) TritonX-100. Once linkage to a specific chromosome had been identified, additional primers were designed flanking tandem repeats and tested for polymorphisms in pooled gDNA samples. Primer sequences of the custom markers 745.25-7 and 594.7-3 are:

745.25-7 F 5′-GCAATGCGTAAGGAATGCTGGGAA-3′

R 5′-GTGTGAGTGTTCTGCTCACGTGTTTG-3′

594.7-3 F 5′-AGTCTGCGTTCATCTTCCACGAGT-3′

R 5′-GCTGGATCTGATGTGGTGTGTTCTGA-3′

Identification of recombinant chromosomes in individual mutant embryos revealed distance and orientation to the mutant loci.

### Molecular Cloning

RNA was extracted from pooled samples of WT or mutant embryos using Trizol reagent according to the manufacturer's protocol (Invitrogen). RNA was resuspended in 1 µL H_2_0/embryo. cDNA was synthesized from 1 µg RNA using the iScript cDNA synthesis kit (Bio-Rad Laboratories). High-fidelity amplification of target genes was performed using KOD enzyme according to manufacturer's protocol (TaKaRa). *utp15* (all isoforms) was cloned into the pCS2 3x FLAG Tag vector with the tag at the N-terminus (pCS2 N FT). After a brief incubation with *Taq* polymerase at 72°, a fragment of *thbs1* was cloned into pCRII TOPO according to the manufacturer's instructions. Sequence analysis of all vectors confirmed presence and orientation of insert.

### In vitro mRNA synthesis and microinjections

mRNA for microinjections was synthesized from 1 µg pCS2 N FT *utp15* plasmids linearized 3′ to the polyA site using the mMessage mMachine Sp6 Kit (Ambion) according to the manufacturer's instructions. RNA concentration was determined by A260:A280 ratio on a SmartSpec3000 Spectrophotometer (Bio-Rad Labortatories). 100 pg of RNA was injected in 1 nL into a minimum of 100 1-cell stage *utp15^LA1908^* embryos per *utp15* isoform.

### qRT-PCR

RNA was extracted from WT and mutant embryos and cDNA synthesized as described above. Relative gene expression was determined by SYBR green based quantitative RT-PCR performed on a BioRad iCycler iQ and analyzed using the BioRad Gene Expression Analysis Macro for Microsoft Excel. Primers were designed using the IDT SciTools online design suite to span exon-exon boundaries where possible and analyzed for minimal secondary structure using the Mfold web server [38]. Primer sequences are as follows:

*Danio rerio gapdh* F 5′- TGTGATGGGAGTCAACCAGGACAA-3′

R 5′- TTAGCCAGAGGAGCCAAGCAGTTA-3′

*Danio rerio utp15* F 5′- CCTGTGCATGTCTGAGTTCAAGCAAC-3′

R 5′- GCCAGACTGAGAATAGATGACGCACA-3′

### Whole-Mount Apoptosis Assay

Apoptosis was visualized in situ using the ApopTag Red in situ cell death detection kit (Millipore), which utilizes Terminal deoxynucleotidyl transferase (TdT) dUTP nick end labeling (TUNEL) chemistry to deposit digoxigenin-labeled nucleotides onto the free 3′-OH termini of cleaved DNA. Embryos were fixed, dehydrated, and digested with Proteinase K as described above. Embryos were then washed in Equilibration Buffer, followed by reaction with TdT. Deposition was stopped in Stop/Wash buffer. Embryos were then blocked in 5% goat serum, 2 mg/mL BSA in PBT. Embryos were stained with the supplied Rhodamine-conjugated anti-digoxigenin antibody diluted in Millipore Blocking Solution according to manufacturer's protocol and digoxigenin-positive nuclei were detected by confocal microscopy and counted in Adobe Photoshop CS4 using the Count Tool.

## Supporting Information

Figure S1 **Gene expression in** ***LA1908*** **is not globally disrupted.** A–D, Markers of neural (*krox20*, A, B) and hematopoietic (*cmyb*, C, D) differentiation were indistinguishable between wild type and mutant embryos. n = 20 embryos for each condition. E–F, Development was not generally delayed, as somite number was not significantly different between *LA1908* mutant and wild type siblings (E). Rather, delay was specific to vascular tissues, exemplified by decreased ISV number in mutant versus wild type embryos at 21 hpf (F). Ten embryos were analyzed per condition. NS = not significantly different, * = p<0.00005.
(TIF)

Figure S2 **ISVs of both arterial and venous origin are present in** ***LA1908*** **mutant embryos.** A–B, Confocal z-stack projection images of Tg[*kdrl*:GFP]^LA1908^ wildtype (A) or mutant (B) embryos. A’–B’’, Single 2.75 µm z-slices of the region outlined in yellow in (A, B), revealing the axial vessel origin of ISVs in day 2 embryos. A’–B’, ISVs originating from the DA are indicated by a red “A”. A’’–B’’, ISVs originating from the PCV are indicated by a blue “V”. Scale bars are 100 µm.
(TIF)

Figure S3 **Expression of growth factors is unchanged in** ***LA1908*** **mutant embryos.** Expression of *bmp2b* (A–D), *bmp4* (E–H), and *vegfaa* (I–L) is indistinguishable between stage-matched wild type (A, C, E, G, I, K) and mutant (B, D, F, H, J, L) embryos. Furthermore, expression of *vegfaa*, the pro-angiogenic signal for ISV angiogenesis, persists in the ventral somites through the time-point after which apoptosis has resolved in *LA1908* mutant embryos (K, L), whereas putative CVP pro-angiogenic signals in the ventral tail, *bmp2b* and *bmp4*, are absent from the ventral tail after one day of development (C, D, G, H), possibly explaining the differential ability of CVP versus ISV angiogenesis to recover after initial delay.
(TIF)

Figure S4 **Induction of p53 mRNA expression in** ***LA1908*** **mutant embryos.** A–B, Expression of p53 mRNA is strongly induced by loss of utp15. Upregulation is observed in all tissues in mutant (B) relative to wildtype (A) embryos. C–D, Vibratome cross-section of embryos shown in Figure 6 F–G, revealing induction of *thbs1*, particularly in the dorsolateral roof and floor plate of the neural tube.
(TIF)

Movie S1 **CVP sprouting is active between 25 and 28 hpf in wildtype embryos.** Time-lapse confocal images of wild type embryos in Tg[*kdrl*:GFP] genetic background from 25–28 hpf. Venous endothelial sprouts are clearly evident during CVP development in WT embryos, maturing into ventrally migrating tip cells to form the primitive plexus.
(WMV)

Movie S2 **CVP sprouting is defective in** ***LA1908*** **mutant embryos.** Time-lapse confocal images of *LA1908* mutant embryos in Tg[*kdrl*:GFP] genetic background from 25–28 hpf. Transient venous endothelial sprouts were formed, but quickly retracted and rarely matured into ventrally migrating tip cells (indicated by arrows).
(WMV)
