# Supplementary figures and images for: Mutation in utp15 Disrupts Vascular Patterning in a p53-Dependent Manner in Zebrafish Embryos
Source: PLoS One. 2011 Sep 20;6(9):e25013. doi: 10.1371/journal.pone.0025013 (PMC3176792; doi:10.1371/journal.pone.0025013)

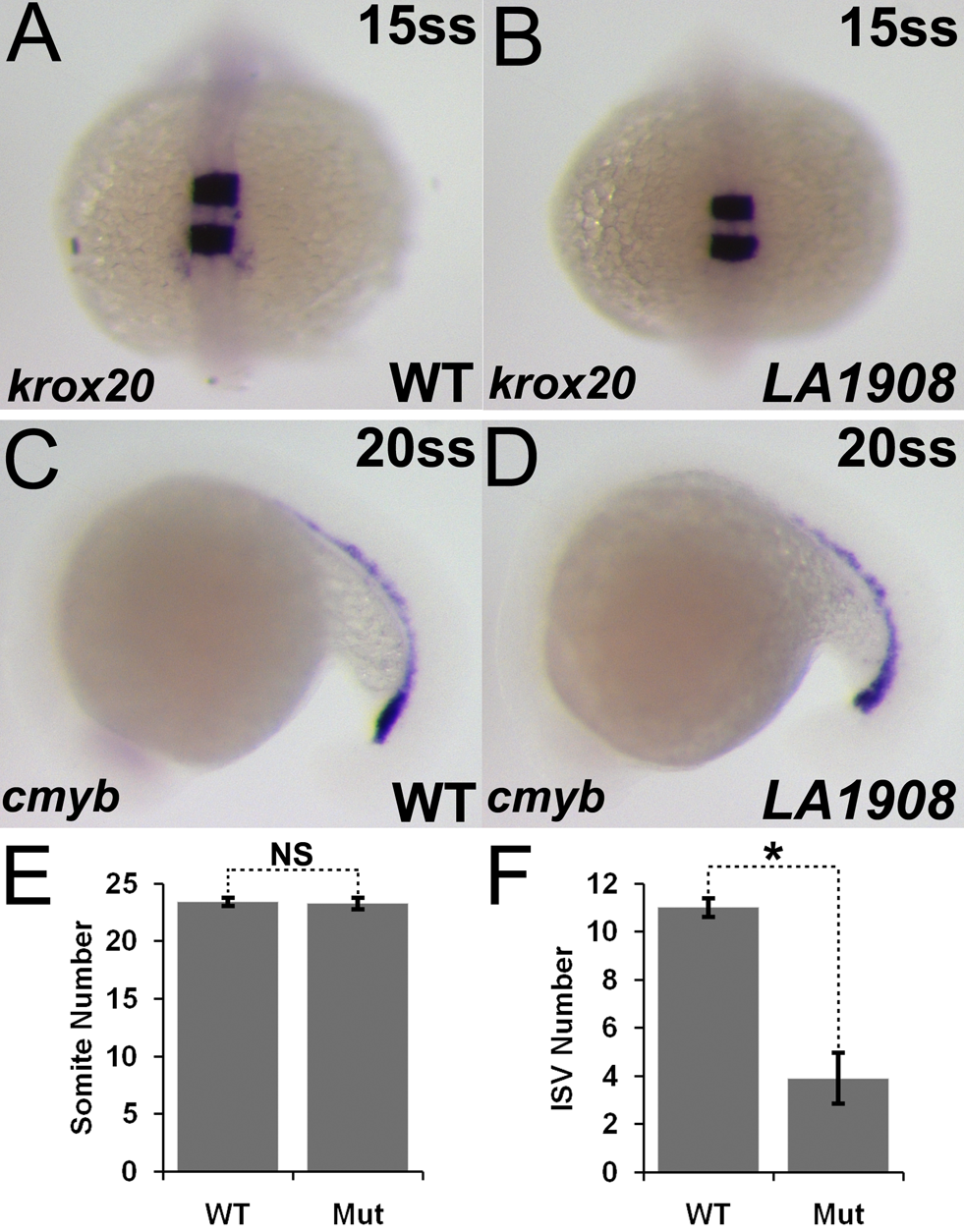

Supplement: Figure S1 — Gene expression in LA1908 is not globally disrupted. A–D, Markers of neural (krox20, A, B) and hematopoietic (cmyb, C, D) differentiation were indistinguishable between wild type and mutant embryos. n = 20 embryos for each condition. E–F, Development was not generally delayed, as somite number was not significantly different between LA1908 mutant and wild type siblings (E). Rather, delay was specific to vascular tissues, exemplified by decreased ISV number in mutant versus wild type embryos at 21 hpf (F). Ten embryos were analyzed per condition. NS = not significantly different, * = p<0.00005. (TIF) [file pone.0025013.s001.tif]

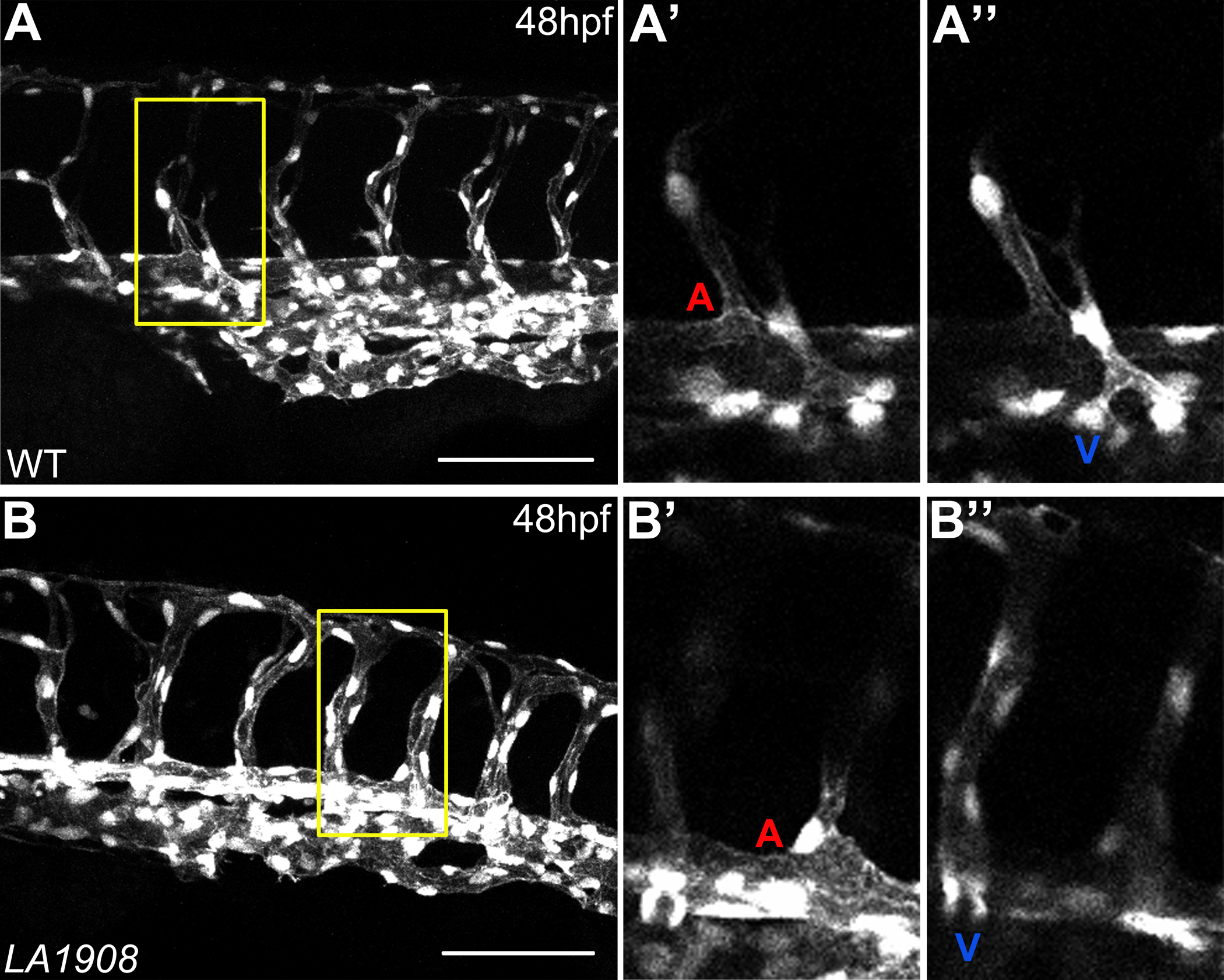

Supplement: Figure S2 — ISVs of both arterial and venous origin are present in LA1908 mutant embryos. A–B, Confocal z-stack projection images of Tg[kdrl:GFP]LA1908 wildtype (A) or mutant (B) embryos. A’–B’’, Single 2.75 µm z-slices of the region outlined in yellow in (A, B), revealing the axial vessel origin of ISVs in day 2 embryos. A’–B’, ISVs originating from the DA are indicated by a red “A”. A’’–B’’, ISVs originating from the PCV are indicated by a blue “V”. Scale bars are 100 µm. (TIF) [file pone.0025013.s002.tif]

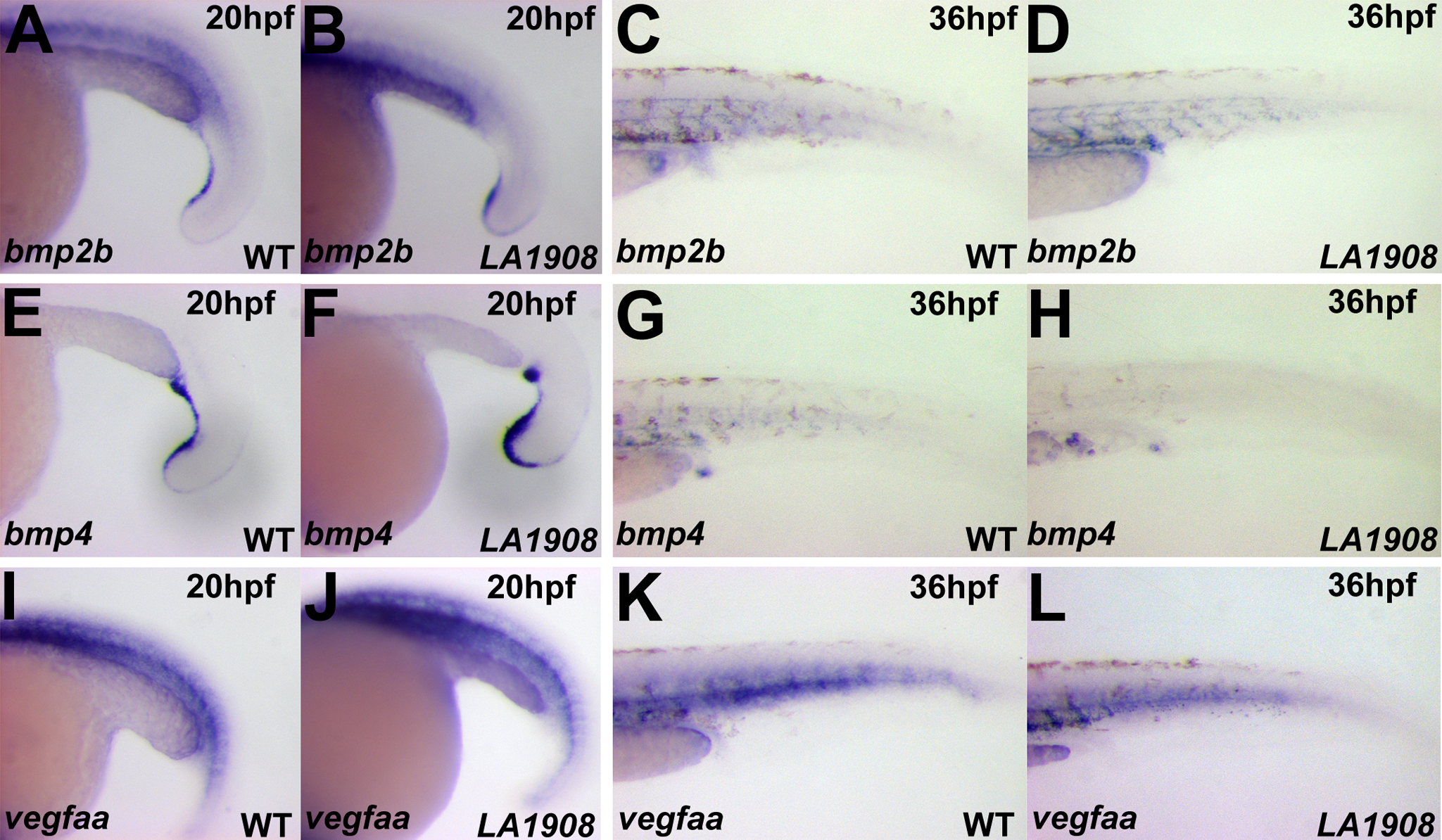

Supplement: Figure S3 — Expression of growth factors is unchanged in LA1908 mutant embryos. Expression of bmp2b (A–D), bmp4 (E–H), and vegfaa (I–L) is indistinguishable between stage-matched wild type (A, C, E, G, I, K) and mutant (B, D, F, H, J, L) embryos. Furthermore, expression of vegfaa, the pro-angiogenic signal for ISV angiogenesis, persists in the ventral somites through the time-point after which apoptosis has resolved in LA1908 mutant embryos (K, L), whereas putative CVP pro-angiogenic signals in the ventral tail, bmp2b and bmp4, are absent from the ventral tail after one day of development (C, D, G, H), possibly explaining the differential ability of CVP versus ISV angiogenesis to recover after initial delay. (TIF) [file pone.0025013.s003.tif]

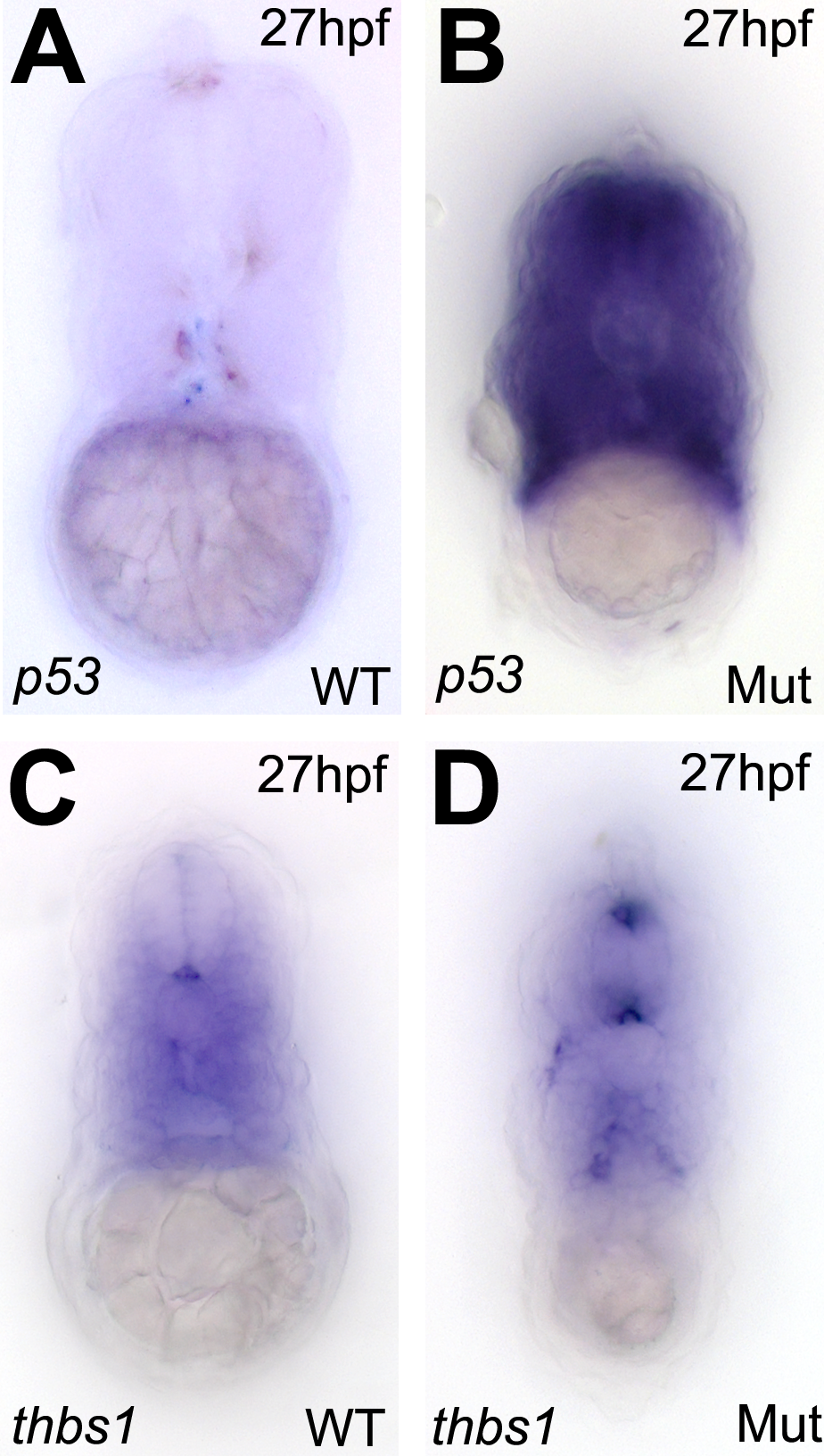

Supplement: Figure S4 — Induction of p53 mRNA expression in LA1908 mutant embryos. A–B, Expression of p53 mRNA is strongly induced by loss of utp15. Upregulation is observed in all tissues in mutant (B) relative to wildtype (A) embryos. C–D, Vibratome cross-section of embryos shown in Figure 6 F–G, revealing induction of thbs1, particularly in the dorsolateral roof and floor plate of the neural tube. (TIF) [file pone.0025013.s004.tif]
